# Active and diverse viruses persist in the deep sub-seafloor sediments over thousands of years

**DOI:** 10.1038/s41396-019-0397-9

**Published:** 2019-03-15

**Authors:** Lanlan Cai, Bo B. Jørgensen, Curtis A. Suttle, Maoqiu He, Barry A. Cragg, Nianzhi Jiao, Rui Zhang

**Affiliations:** 1 State Key Laboratory of Marine Environmental Science, College of Ocean and Earth Sciences, Xiamen University (Xiang’an), Xiamen, Fujian China; 2 Institute of Marine Microbes and Ecospheres, Xiamen University (Xiang’an), Xiamen, Fujian China; 3 Center for Geomicrobiology, Department of Bioscience, Aarhus University, 8000 Aarhus C, Denmark; 4 Departments of Earth, Ocean and Atmospheric Sciences, Microbiology and Immunology, and Botany, The Institute for the Oceans and Fisheries, University of British Columbia, Vancouver, BC Canada; 5 State Key Laboratory of Trophic Oceanography, South China Sea Institute of Oceanology, Chinese Academy of Sciences, Guangzhou, China; 6 School of Earth and Ocean Sciences, Cardiff University, Main Building, Park Place, Cardiff, United Kingdom

**Keywords:** Microbial ecology, Biodiversity

## Abstract

Viruses are ubiquitous and cause significant mortality in marine bacterial and archaeal communities. Little is known about the role of viruses in the sub-seafloor biosphere, which hosts a large fraction of all microbes on Earth. We quantified and characterized viruses in sediments from the Baltic Sea. The results show that the Baltic Sea sub-seafloor biosphere harbors highly abundant viruses with densities up to 1.8 × 10^10^ viruses cm^−3^. High potential viral production down to 37 meters below seafloor in ca. 6000-years-old sediments and infected prokaryotic cells visible by transmission electron microscopy demonstrate active viral infection. Morphological and molecular data indicate that the highly diverse community of viruses includes both allochthonous input from the overlying seawater and autochthonous production. The detection of cyanophage-like sequences showed that viruses of phototrophic hosts may persist in marine sediments for thousands of years. Our results imply that viruses influence sub-seafloor microbial community dynamics and thereby affect biogeochemical processes in the sub-seafloor biosphere.

## Introduction

The deep biosphere extends hundreds to thousands of meters below the seafloor [1–4]. It harbors by far the largest reservoir of organic carbon on Earth and is inhabited by an estimated number of 3 × 10^29^ prokaryotic cells, or half of all prokaryotes in the ocean [5]. Highly diversified members of the Bacteria and Archaea, mostly without cultivated relatives, have been revealed by recent molecular ecological studies [2, 3]. The sub-seafloor microbial communities are responsible for the degradation of organic matter buried in sub-seafloor sediments, though the mean metabolic activity per prokaryotic cell is low [2, 4]. Despite the ecological and biogeochemical importance of prokaryotes in the deep biosphere, there is little information about their mortality and the regulation of their turnover. Since eukaryotic grazers are rare or absent in deep sediments, viral lysis is likely the most important cause of prokaryotic mortality [6, 7].

It is well known that viruses are abundant in surface sediments and cause mortality of bacteria and archaea, hence affecting benthic microbial processes and nutrient cycling [6, 8]. Viruses also occur hundreds of meters below the seafloor, where their abundances exceed those of their putative prokaryotic hosts [9–11]. The total, global abundance of viruses in the sub-seafloor has been estimated to be 3.5 × 10^31^, i.e., a hundred-fold higher than the total microbial cell number. These viruses represent a large proportion of the virus particles estimated to occur on Earth [12]. Moreover, indirect evidences such as high virus-to-cell ratios and the expression of viral homologs in metatranscriptomes, suggest ongoing production of viruses in deep sediments with consequent ecological and biological effects [9, 13]. However, there are few data on virus diversity in sub-seafloor sediments [13], and there are very few experimental measurements examining the function of viruses in the sub-seafloor.

The Baltic Sea is one of the world’s largest intracontinental brackish basins, characterized by high sedimentation rates and high concentrations of organic matter and nutrients that fuel the growth of benthic microbial communities. The deepest Baltic sedimentary record to date was recovered during the Integrated Ocean Drilling Project (IODP) Expedition 347 which captured the transition from the glacio-lacustrine clay of the Baltic Ice Lake more than 8000 years ago to the organic-rich mud of the modern Baltic Sea [14]. We studied the abundance, the lytic and inducible lysogenic production, and the morphological and phylogenetic diversity of viruses in the brackish-marine sediments of the Baltic Sea (Fig. S1). Our data provide evidence that viruses are a diverse and potentially highly active component of the ecosystem in sub-seafloor sediments of the Baltic Sea.

## Results

### Population size, activity, and life strategy of deep viruses

**Fig. 1:**
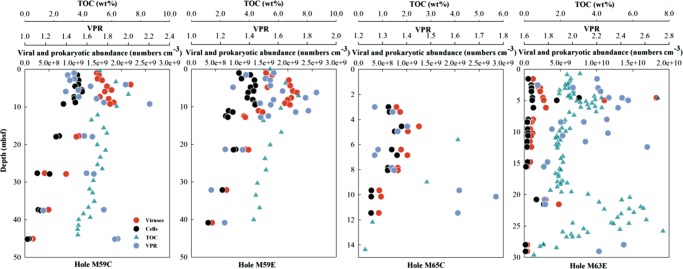
Depth profiles of viral abundance, prokaryotic abundance, virus-to-prokaryote ratio (VPR) and total organic carbon (TOC) in sediment cores from four Baltic Sea holes. The data for TOC is from ref. ^14^. Note different scales

Viral counts were very similar among four drilled holes from three sites in the Baltic Sea (Fig. 1, Fig. S2, see Supplementary Methods for details). From depths of 1 to 45 m below seafloor (mbsf) with ages upto ca. 7000 years [15], viral abundances varied from (9.6 ± 0.2) × 10^7^ to (1.8 ± 0.1) × 10^10^ particles cm^−3^ and were usually higher than prokaryotic abundances, (5.1 ± 0.1) × 10^7^ to (7.6 ± 0.2) × 10^9^ cells cm^−3^. As a general trend for all four holes, viral abundances increased with depth beneath the surface and peaked at 4–5 mbsf, where a peak in total organic carbon (TOC) occurred, followed by a steady decline with depth. Similar patterns were observed for microbial cell abundances (Fig. 1).

**Fig. 2:**
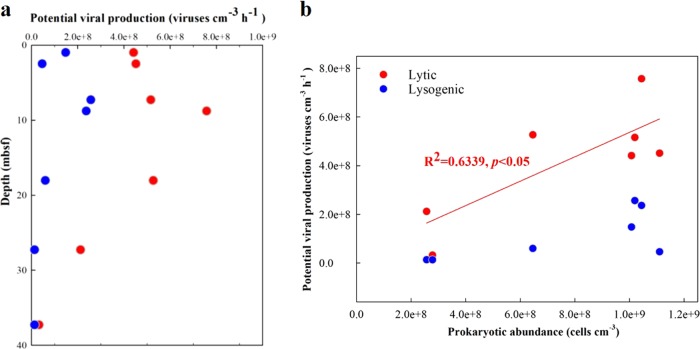
Apparent production of viruses in the sediments. (**a**), Depth profiles of apparent lytic (red circles) and inducible lysogenic (blue circles) viral production in the sediments at Hole M59C. (**b**), Relationships between lytic and lysogenic viral production and prokaryotic abundance. The red line represents the linear regression between lytic viral production and prokaryotic abundance

The apparent lytic viral production, estimated by dilution method (Supplementary Methods, Fig. S3), in the top 0–20 mbsf was very high, with lysis rates ranging from 4.4 × 10^8^ to 7.6 × 10^8^ viruses cm^−3^ h^−1^, and lysogenic viral production, estimated by mitomycin C induction, ranged from 4.6 × 10^7^ to 2.6 × 10^8^ viruses cm^−3^ h^−1^. At 37 mbsf, the age of which was ca. 6000 years [15], the apparent lytic viral production had decreased to 3.2 × 10^7^ viruses cm^−3^ h^−1^ (Fig. 2a). Across all depths there was a significant positive correlation between apparent lytic viral production and prokaryotic cell abundance (Fig. 2b). This suggests that viral replication depends on host density. Recent investigations have reported that lysogens (prokaryotic cells containing prophages) comprise a significant part of the heterotrophic microbial population and that lysogenic infection may be common in environments that may be unfavorable for viral survival, such as deep sediments [13]. In the sediments of the Baltic Sea, inducible lysogenic viral production accounted for 6 to 33% (average 19%) of the total potential viral production, indicating that lysogenic infection was an important, but not a predominant, life cycle of viruses in the sediments.

### Morphological and genetic diversity of sub-seafloor viruses

**Fig. 3:**
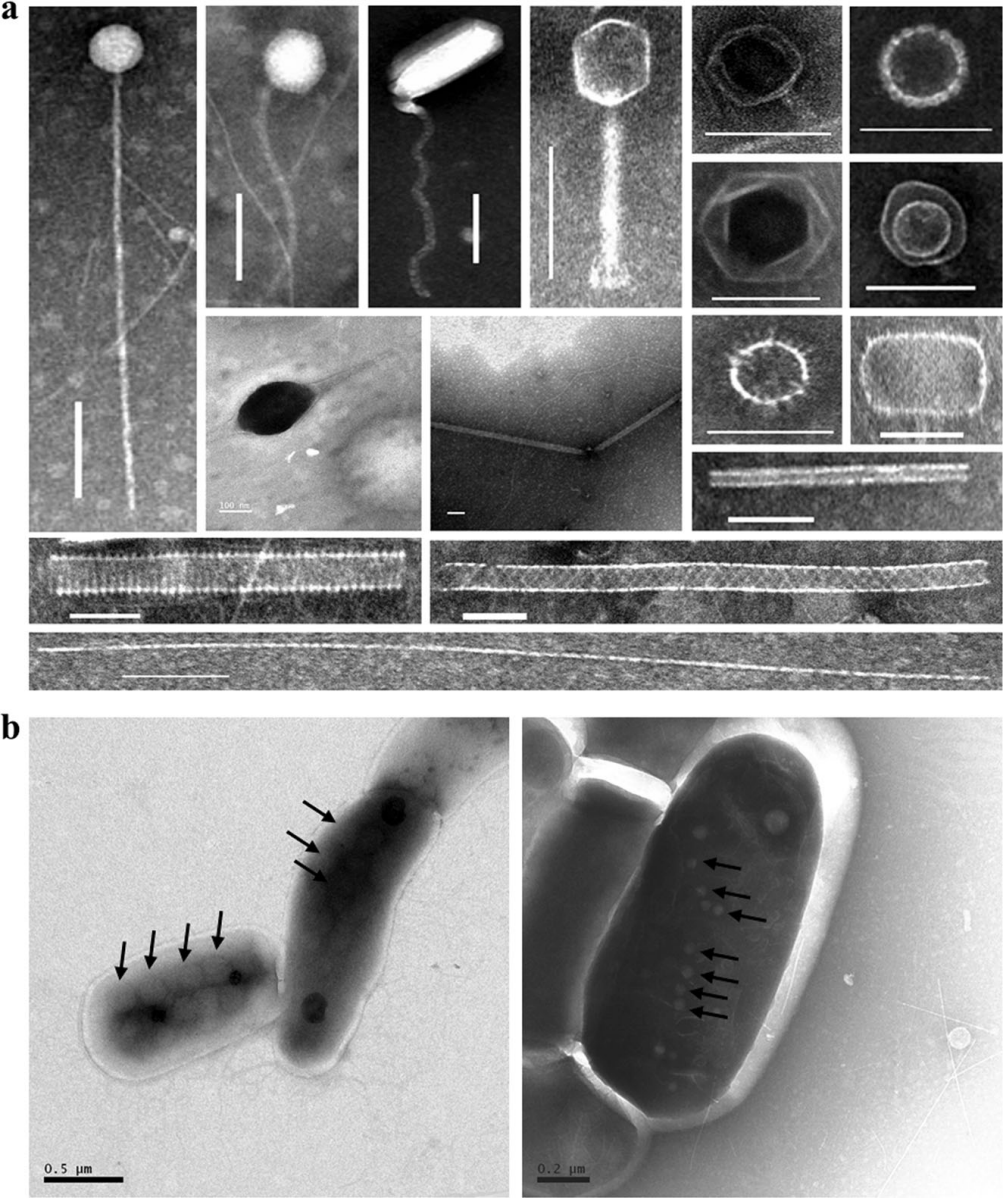
Transmission electron micrographs showing the morphologies of virus-like particles and infected cells in the deep sediments of the Baltic Sea. (**a**), Examples of virus-like particles observed. Scale bars: 100 nm. (**b**), Infective viruses (arrows) in the visibly infected cells. Samples were recovered from deep sediments down to 70 mbsf in Hole M59C

Three tailed virus-like morphotypes were observed at all depths, corresponding to members of the *Siphoviridae* (long, flexible non-contractile tails), *Podoviridae* (short, non-contractile tails), and *Myoviridae* (contractile tails), as well as icosahedral particles without tails (Fig. 3a, Fig. S4, Supplementary Methods). A large number of filamentous, spherical, encapsulated, rod-shaped and spindle-shaped virus-like particles (VLPs) were also detected, especially in the deeper sediment. Importantly, we observed intact VLPs in deep sub-seafloor prokaryotic cells for the first time (Fig. 3b), thus demonstrating the in situ assembly of viral particles in the hosts.

**Fig. 4:**
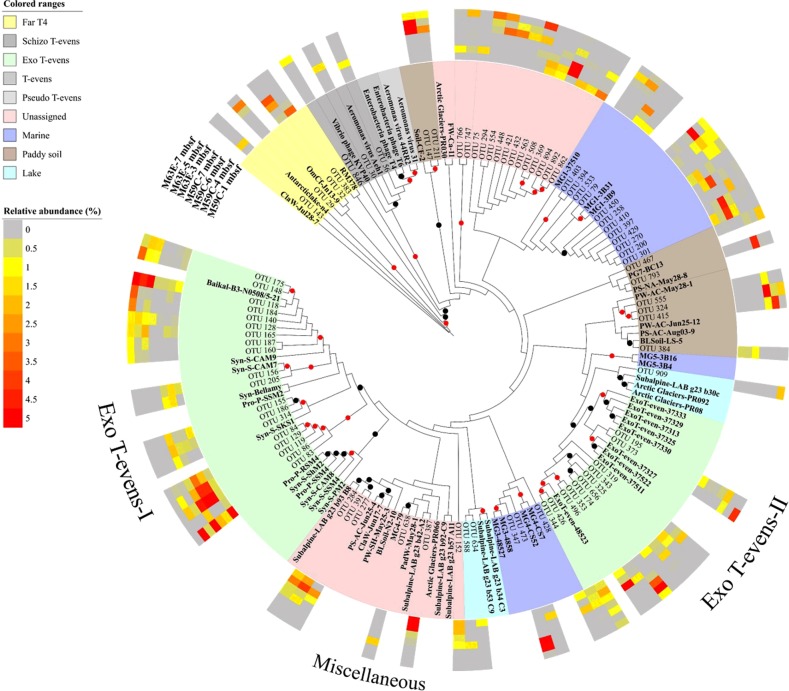
Maximum-likelihood phylogenetic analysis based on major *g23* OTUs of amino acid sequences. OTUs with relative abundances >1% in each sample (a total of 84 OTUs) from Holes M59C and M63E of the Baltic Sea sediment were selected. Different colored ranges indicate *g23* sequences from different groups or origins. Reference sequences are in bold text. Black and red dots show internal nodes with a >50% and >80% bootstrap (1000-fold replicated) support, respectively. The outer colored rings indicate the relative abundance of sequences of each OTU in each sample. Light gray indicates sequences undetected in the samples (relative abundance of 0%)

Genetic diversity in the deep virosphere of the Baltic Sea was demonstrated by sequence analysis of the major capsid gene (*g23*) of T4-like myoviruses, one of the most abundant, ubiquitous, and well-studied virus groups in the environment. Hierarchical cluster analysis based on a Bray–Curtis dissimilarity matrix showed that the composition of viral assemblages differed significantly between sites (*p* < 0.05, Fig. S5, Table S1). This might result from the geographic and environmental differences between these holes, where M59C is located in the southern Little Belt with a high sedimentation rate, resulting in a complex and diverse viral community, while M63E is situated in the central part of the anoxic Landsort Deep, the deepest basin in Baltic Sea [14]. A BIO-ENV test indicated that the genetic composition was correlated with the total organic carbon in the sediment, and to a greater extent with other environmental parameters of interstitial water such as salinity and the concentrations of rubidium (Rb), lithium (Li^+^), calcium (Ca^2+^) (Table S2). The identified 84 major operational taxonomic units (OTUs) with relative abundances >1% in each sample showed 52–99% identity with sequences in the NCBI non-redundant protein database. They were widely distributed among phylogenetic clusters with related sequences originating from different ecosystems (Fig. 4). A significant portion of these OTUs (36%) was related to two groups of Exo-T-evens viruses infecting marine and freshwater cyanobacteria. Exo-T-evens-I phages were more closely related to the *g23* sequences of cyanophage isolates and environmental sequences recovered from freshwater lakes. The sequences of Exo-T-evens-II group are derived from environmental-only sequences from marine environments. Overall, phylogenetic analysis based on the g*23* gene indicated that diverse T4-like phage groups are preserved in the Baltic Sea sub-seafloor sediments.

## Discussion

Deep sub-seafloor sediments harbor large numbers of prokaryotes with high genetic and metabolic diversity, yet the controls on their population size and diversity are unclear. The low bioavailability of organic matter suggests a bottom-up control of their population size, which is supported by extremely low metabolic rates [4, 16]. Our data suggest that in the deep biosphere, where metazoan and protozoan grazing is thought to be absent, viral lysis may be a major top-down factor in controlling prokaryotic population size, turnover rate, and diversity.

**Fig. 5:**
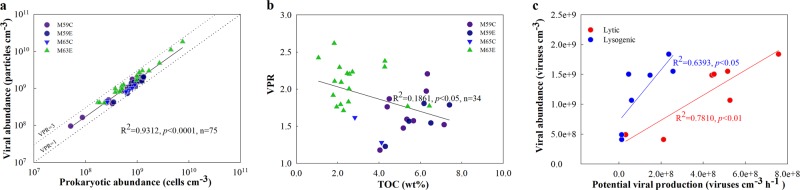
Relationships between (**a**) viral abundance and prokaryotic abundance, (**b**) virus to prokaryote ratio (VPR) and total organic carbon (TOC), and (**c**) viral abundance and potential lytic or inducible lysogenic viral production

The viral and prokaryotic abundances in the Baltic Sea sub-seafloor sediments are similar to those in marine surface sediments [8, 17] or in other sub-seafloor sediments in productive regions where microbial activity is enhanced by high concentrations of organic matter [11]. The abundances are much higher than in deep-sea surface and sub-seafloor sediments in oligotrophic regions [7, 9, 10, 18, 19]. The significant and positive relationship between viral abundance and prokaryotic abundance for all four holes (*p* < 0.0001, Fig. 5a) is consistent with observations from different marine waters [20–22], and has been found to apply over seven orders of magnitude world-wide for diverse sediments with wide ranging organic matter content [9–11, 19]. These data are consistent with the view that there are broad relationships across systems between the abundances of viruses and the prokaryotes that are their putative hosts, although the abundance ratios vary across systems [20].

Virus-to-prokaryote ratios (VPRs) varied only from 1.1 to 2.6 (Fig. 1), and were in the range reported for the deep sub-seafloor seabed of Saanich Inlet [11], the eastern margin of the Porcupine Seabight [18], the Peru margin, and the Bering Sea [9], as well as lake and coastal surface sediments (e.g., refs. 23 and 24). Even though the total abundances of viruses and cells declined by two orders of magnitude, the small variation in VPRs across depths suggests a stable balance between the production and removal of viruses and their hosts. Nonetheless, higher VPRs were observed in deeper sediments with fewer cells and lower organic matter content (Fig. 5b), suggesting better preservation and lower decay rates for viral particles at depth. Low organic matter availability and small prokaryotic population sizes would mean less overall metabolic activity and, thus, likely less degradation of viral particles. At the same time, higher clay content with high surface charge and diminishing pore spaces [14] may increase the absorbance of viral particles onto sediment particles, providing further protection of viruses from degradation [25].

The positive correlation between viral abundance and apparent viral production rate (Fig. 5c) indicates that the population size of the deep viruses was, at least partially, controlled by an autochthonous production of new viruses. TEM analyses (Fig. 3) showed the presence of VLPs inside microbial cells in the deep sediment (down to 70 mbsf, >7 ka old), providing evidence that viruses are produced in indigenous microbial hosts. The observation of particular VLP morphologies (such as filamentous, spherical, encapsulated, rod-shape, and spindle-shape), rarely found in the water column, suggests that potentially sediment-specific viral morphotypes were produced in situ. Furthermore, the presence of unassigned clusters of *g23* sequences should also be an indication of autochthonous production by infecting hosts unique to the sub-seafloor sediments. If we assume a burst size (the number of viral particles released from each cell upon lysis) of 45, which is a mean value obtained from a global survey of surface sediments [8], our lytic viral production experiments suggest a mean viral turnover rate of 0.39 h^−1^ (0.30–0.52 h^−1^) for the upper 30 mbsf (Table 1). Such rates are similar to those found in high-nutrient surface sediments [8]. However, these rates should be considered only as potential rates as they are too high to be supported by cell biomass turnover in sub-seafloor sediments.

**Table 1:** Viral turnover rate (V_TR_), viral turnover time (V_TT_), virus-mediated prokaryotic mortality rate (VMM), percentage of prokaryotic cells lysed by viruses per hour, virus-induced prokaryotic turnover time (VP_TR_), and rate of carbon release by lysis in the sediments of Hole M59C. VMM was calculated by dividing lytic viral production by a mean burst size of 45, a mean number obtained from a global survey of surface sediments

Depth (mbsf)	V_TR_ (h^−1^)	V_TT_ (h)	VMM (Cells·cm^−3^·h^−1^)	Percentage of cells lysed (% h^−1^)	VP_TR_ (d)	Carbon released (μg·cm^−3^·d^−1^)
0.98	0.30	3.37	9.8E + 06	0.97	4.28	0.47
2.48	0.30	3.33	1.0E + 07	0.99	4.22	0.48
7.28	0.33	3.01	1.1E + 07	1.12	3.71	0.55
8.78	0.41	2.43	1.6E + 07	1.61	2.58	0.81
18.03	0.49	2.03	1.1E + 07	1.81	2.30	0.56
27.28	0.52	1.93	4.7E + 06	1.83	2.27	0.23
37.28	0.07	15.27	7.1E + 05	0.26	16.24	0.03

Note: Source Ref. [8]. The percentage of cells lysed was determined by dividing VMM by prokaryotic cell abundance. Carbon released by viral lysis = VMM × cell carbon content, and prokaryotic carbon content were based on an estimate of 20 fg C cell^−^^1^ in the sub-seafloor sediments of Baltic Sea [8]. Note that the data represent potential rates obtained from incubation experiments and exceed the natural rates

Another potential source of sub-seafloor viruses in the Baltic Sea is allochthonous inputs from nutrient-rich and fast-sinking detritus from freshwater discharge and phytoplankton blooms [26]. For example, 48% of *g23* sequences were assigned to cyanophages-like sequences (Fig. S6). This is not unexpected given that cyanobacteria bloom every summer and sink to the sediment in the Baltic Sea. This is evidenced by the presence of cyanobacterial sequences in metagenomes from sediments retrieved from the same site (M0059) [27]. The clustering of OTUs with sequences from freshwater and soil environments further indicate allochthonous contributions of viruses from surrounding environments. In addition, TEM analyses revealed a significant proportion of non-tailed viruses, similar to nuclear cytoplasmic large DNA viruses (NCLDVs), which appear to be members of the family *Phycodnaviridae* infecting eukaryotic phytoplankton [28].

High-viral production detected in the sediments suggests that viruses cause significant bacterial mortality. The calculated potential virus-mediated prokaryotic mortality was 0.5–1.6 × 10^7^ cells cm^−3^ h^−1^ for the upper 30 mbsf and decreased to 7.1 × 10^5^ cells cm^−3^ h^−1^ below 30 mbsf (Table 1). This implies prokaryotic mortality rates of 1.0 to 1.8% h^−1^ that increased from the sediment surface to 27 mbsf, and then dropped to their lowest value at 37 mbsf. The calculated turnover times of prokaryotes induced by the potential virus lysis rates ranged from 2.3 to 16.2 days and were similar to results from studies of deep-sea surface sediments (<2 to 67 days) [17, 29], but were much faster than those reported for the deep biosphere [30]. The higher rates that we measured are presumably due to a stimulated release of viral particles by the manipulations used in the incubation experiments. Nevertheless, our data demonstrated a high potential for viral lysis in the deep biosphere.

Viral lysis of prokaryotic cells releases organic material, which supports growth and nutrient recycling by non-infected cells [18, 31, 32]. With a mean cellular biomass of 20 fg C cell^−1^ for prokaryotic assemblages in the sediments [8], our calculation of carbon released by potential viral lysis in the Baltic Sea sediments ranges from 0.03 to 0.81 μg cm^−3^ d^−1^. Previous studies showed that the majority of dissolved organic carbon in the sediment was recalcitrant and cannot be easily utilized by microbes [2]. Although the amount of carbon released by viral lysis is small relative to the total organic matter degraded by microorganisms, its bio-availability is high [33, 34]. Ultimately, virus recycled organic carbon may support a part of the bacteria production in the deep biosphere.

Overall, this study demonstrates that viruses are abundant, diverse, and likely active members of sub-seafloor Baltic Sea sediments. On average, there are billions of viral particles per cm^3^ of sediment, which display high morphological diversity based on TEM, and high genetic diversity based on sequence analysis of *g23* as a marker gene for T4-like phages. Our analysis of the sequences also suggests that some viruses in the deep sediments originated from the overlying water column. The detection of cyanophage-like sequences in the Baltic Sea sediments indicates long-term virus preservation. High potential viral production and visibly infected prokaryotic cells provide strong evidence that viral infection is ongoing and is a potential source of microbial mortality in the deep biosphere. This is of particular importance in an environment in which other direct sources of microbial mortality may be absent or rare. Taken together, our results show that viruses are an abundant, active, and diverse component of the deep biosphere with important potential influence on microbial ecology and biogeochemical cycling.

## Supplementary information

Supplemental information
