# Supplementary material for: Active and diverse viruses persist in the deep sub-seafloor sediments over thousands of years
Source: ISME J. 2019 Mar 15;13(7):1857–64. doi: 10.1038/s41396-019-0397-9 (PMC6776017; doi:10.1038/s41396-019-0397-9)
Supplement: Supplementary file 1 — Supplemental information [file 41396_2019_397_MOESM1_ESM.docx]

Supplementary Information for

Active and diverse viruses persist in deep sub-seafloor sediments over thousands of years

Lanlan Cai, Bo B. Jørgensen, Curtis A. Suttle, Maoqiu He, Barry A. Cragg, Nianzhi Jiao, Rui Zhang

Corresponding authors: Rui Zhang, Nianzhi Jiao

Emails: ruizhang@xmu.edu.cn, jiao@xmu.edu.cn

**This file includes:**

Materials and Methods

Figures S1 to S6

Tables S1 to S2

**Materials and Methods**

**Sample collection**

Sediments were sampled from four boreholes: M59C and M59E in southern Little Belt, M63E in the Landsort Deep and M65C in the Bornholm Basin during the IODP Expedition 347 in the Baltic Sea in 2013 (Fig. S1). The Baltic Sea is an organic-rich basin with a high sedimentation rate of 100-500 cm ky^-1^ [1]. Water depths at the sampling sites ranged between 37.1 and 437.1 m. The physical and chemical parameters of sediment and pore water (such as TOC, TIC, pH, inorganic nutrients, metal concentrations) were described in IODP 347 Expedition Reports [1] and are available online (<http://publications.iodp.org/proceedings/347/347toc.htm>). Samples for viral and microbial counts were collected from the central part of whole-round piston cores with sterile cut-off 10 mL syringes and stored at −80 °C until analysis. Whole-round core samples (5 cm long) for viral production experiments were collected and anaerobically stored with N_2_ gas at 4 °C. For molecular analysis, whole-round core samples were frozen immediately at −80 °C.

**Enumeration of viruses and prokaryotic cells**

To estimate the number of viruses and prokaryotic cells in the sediments, triple 1 cm^3^ samples of frozen sediment were transferred into sterile 50 mL centrifuge tubes and promptly fixed in a final concentration of 0.5% glutaraldehyde, prepared in 9 mL 0.02 μm pore-size filtered SM buffer (50 mM Tris-HCl pH 7.5, 0.1 M NaCl, 8 mM MgSO_4_). The sediments were homogenized by vortexing for 15 min in the dark and then incubated in 5 mM (final concentration) sodium pyrophosphate solution (0.02 μm filtered) to promote the separation of viruses and prokaryotes from the sediment particles. The slurries were then sonicated at 60 W on ice for 3 min with 30 sec manual shaking every min to detach the viruses from sediment matrices [2]. In order to minimize particle masking of viruses and cells during microscopic examination, slurries were diluted to 50 mL with filtered SM buffer. Between 20 and 100 μL of subsample was further diluted in 1 mL SM buffer and filtered onto 0.02 μm pore-size membrane filters (Anodisc 25, Whatman). The filters were stained with SYBR Green I (Molecular Probes) and observed in a fluorescence microscope (BX51, Olympus, Tokyo, Japan) using the WIB fluorescence filter settings (excitation wavelength: 460-490 nm and emission wavelength: > 510 nm). Viruses and cells were manually counted from at least 30 microscopic fields, ensuring a minimum count of 400 for each sample (Fig. S2).

**Viral production**

Apparent viral production in the sediments of Hole M59C was estimated through the dilution technique (Fig. S3) as applied to marine surface sediments [3]. This technique relies on incubations of sediment samples with virus-free seawater, which minimizes the effects of new viral infections, and the impact of protozoa grazing on prokaryotes. It also has the advantage of reducing viral loss due to possible enzymatic degradation during incubation. Viral production values were determined based on the increase in viral number over time determined by epifluorescence microscopy. Briefly, 10 cm^3^ sediment samples were diluted with virus-free surface seawater (1:10 vol/vol) collected from the same station in the Baltic Sea and incubated anaerobically in the dark at 4 °C, in three replicates. Mitomycin C (1 μg/mL final concentration in 0.02-μm prefiltered seawater) was used to estimate the inducible lysogenic viral production. All the procedures of sample preparation and incubation were performed in an anaerobic chamber (Shel Lab, Bactron Ⅲ, USA). A palladium catalyst was used in the chamber to remove any oxygen. Subsamples for viral counts were collected every 3 h to 12 h. Rates of viral production were calculated using the online program VIPCAL (http://www.univie.ac.at/nuhagphp/vipcal) [4] for triplicate incubations.

**Transmission Electron Microscopy (TEM) analysis**

Viral morphology was investigated by TEM after the detachment of viruses from sediment particles. The detachment was performed as above. A further purification step was performed with polyethylene glycol (PEG-8000) (10% w/v) precipitation. Ten μL of viral concentrate was placed on formvar, carbon-coated copper electron microscopy grids (200 mesh) and the viruses allowed to adsorb for 20 min [5]. Virus-like particles were negatively stained with 1% (w/v) phosphotungstic acid for 1 min. Excess stain was removed by filter paper and the grids were air dried prior to examination with a JEM-2100 electron microscope (microscope accelerating voltage 120 kV).

**Viral diversity based on *g23* gene**

Total DNA was extracted from 1 g of sediment samples from holes M59C and M63E using FastDNA SPIN kit for Soil (Qbiogene inc., Carlsbad, CA, USA) according to the manufacturer’s instructions [6, 7]. Primers MZIA1bis and MZIA6 were used to amplify fragments of the *g23* gene from T4-like phages [8], and the amplicons were sequenced using a Roche 454 GS FLX system (Roche 454 Life Sciences, Branford, CT, USA). Chimeras were removed and the sequences were grouped into operational taxonomic units (OTUs) defined by sequence identity of 97%. A total of 3,717 clusters of operational taxonomic units were obtained with between 504 and 840 OTUs per sample. Major OTUs with a relative abundance >1% in each sample (a total of 84 OTUs) were selected to translate into amino acids and then construct a maximum likelihood phylogenetic tree with 1000 bootstraps based on the PROTGAMMA model in RAxML [9]. The tree was viewed and graphically edited using iTOL [10]. Dissimilarity between samples was calculated by the Bray-Curtis analysis [11] based on OTU abundance data. The relationship between the viral genetic composition and environmental variables was explored using the BIOENV procedure [12]. The FASTA file has been submitted to NCBI’s Sequence Read Archive under Bioproject: PRJNA416198.

**References**

1. Andrén T, Jørgensen BB, Cotterill C, the Expedition 347 scientists. Baltic sea paleoenvironment. Proceedings of the Integrated Ocean Drilling Program, Vol 347. 2014.

2. Danovaro R, Middelboe M. Separation of free virus particles from sediments in aquatic systems. Man Aquat Viral Ecol. 2010:74-81.

3. Dell'Anno A, Corinaldesi C, Magagnini M, Danovaro R. Determination of viral production in aquatic sediments using the dilution-based approach. Nat Protoc. 2009;4(7):1013-22.

4. Luef B, Luef F, Peduzzi P. Online program 'VIPCAL' for calculating lytic viral production and lysogenic cells based on a viral reduction approach. Environ Microbiol Rep. 2009;1(1):78-85.

5. Borrel G, Colombet J, Robin A, Lehours AC, Prangishvili D, Sime-Ngando T. Unexpected and novel putative viruses in the sediments of a deep-dark permanently anoxic freshwater habitat. ISME J. 2012;6(11):2119-27.

6. Wang G, Hayashi M, Saito M, Tsuchiya K, Asakawa S, Kimura M. Survey of major capsid genes (*g23*) of T4-type bacteriophages in Japanese paddy field soils. Soil Biol Biochem. 2009;41(1):13-20.

7. He M, Cai L, Zhang C, Jiao N, Zhang R. Phylogenetic diversity of T4-Type phages in sediments from the subtropical Pearl River Estuary. Front Microbiol. 2017;8:897.

8. Filée J, Tetart F, Suttle CA, Krisch HM. Marine T4-type bacteriophages, a ubiquitous component of the dark matter of the biosphere. Proc Natl Acad Sci USA. 2005;102(35):12471-6.

9. Stamatakis A. RAxML version 8: a tool for phylogenetic analysis and post-analysis of large phylogenies. Bioinformatics. 2014;30(9):1312-3.

10. Letunic I, Bork P. Interactive tree of life (iTOL) v3: an online tool for the display and annotation of phylogenetic and other trees. Nucleic Acids Res. 2016;44(W1):W242-W5.

11. Bray JR, Curtis JT. An ordination of the upland forest communities of southern Wisconsin. Ecol Monogr. 1957;27:325-49.

12. Clarke KR, Ainsworth M. A method for linking multivariate community structure to environmental variables. Mar Ecol Prog Ser. 1993;92:205-19.


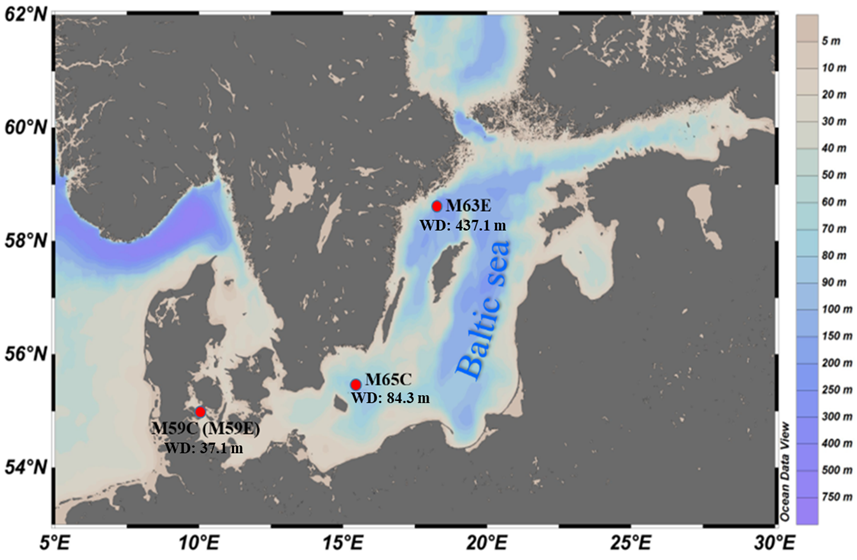


Fig. S1. Coring sites of the IODP drilling expedition 347 with the specific holes sampled for this study. WD: water depth.


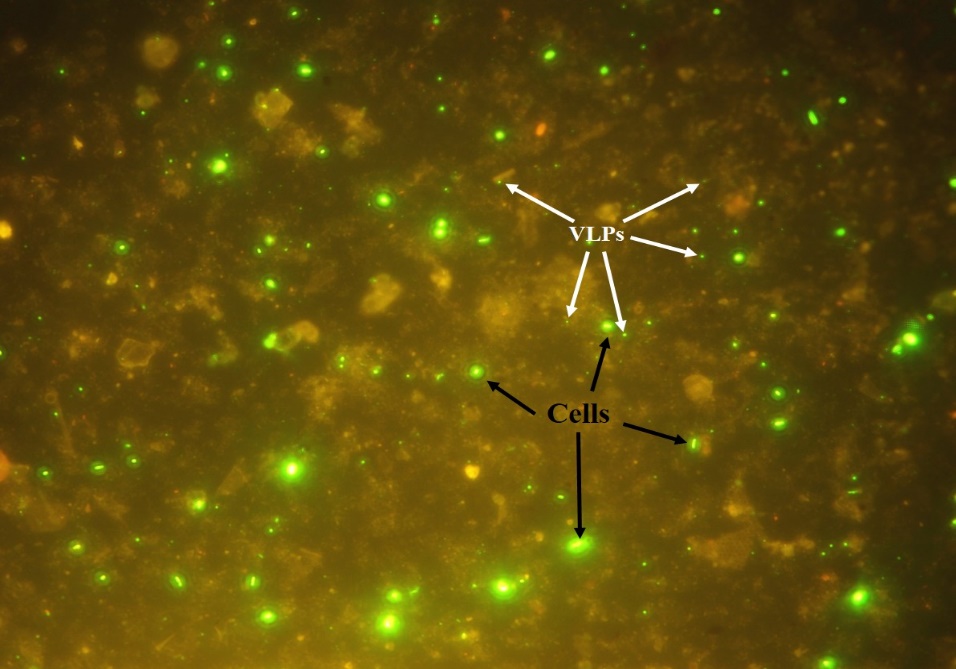


Fig. S2. Epifluorescence micrograph of sediment sample from 6 mbsf of Hole M63E. Black arrows point to prokaryotic cells. White arrows point to virus-like particles (VLPs).


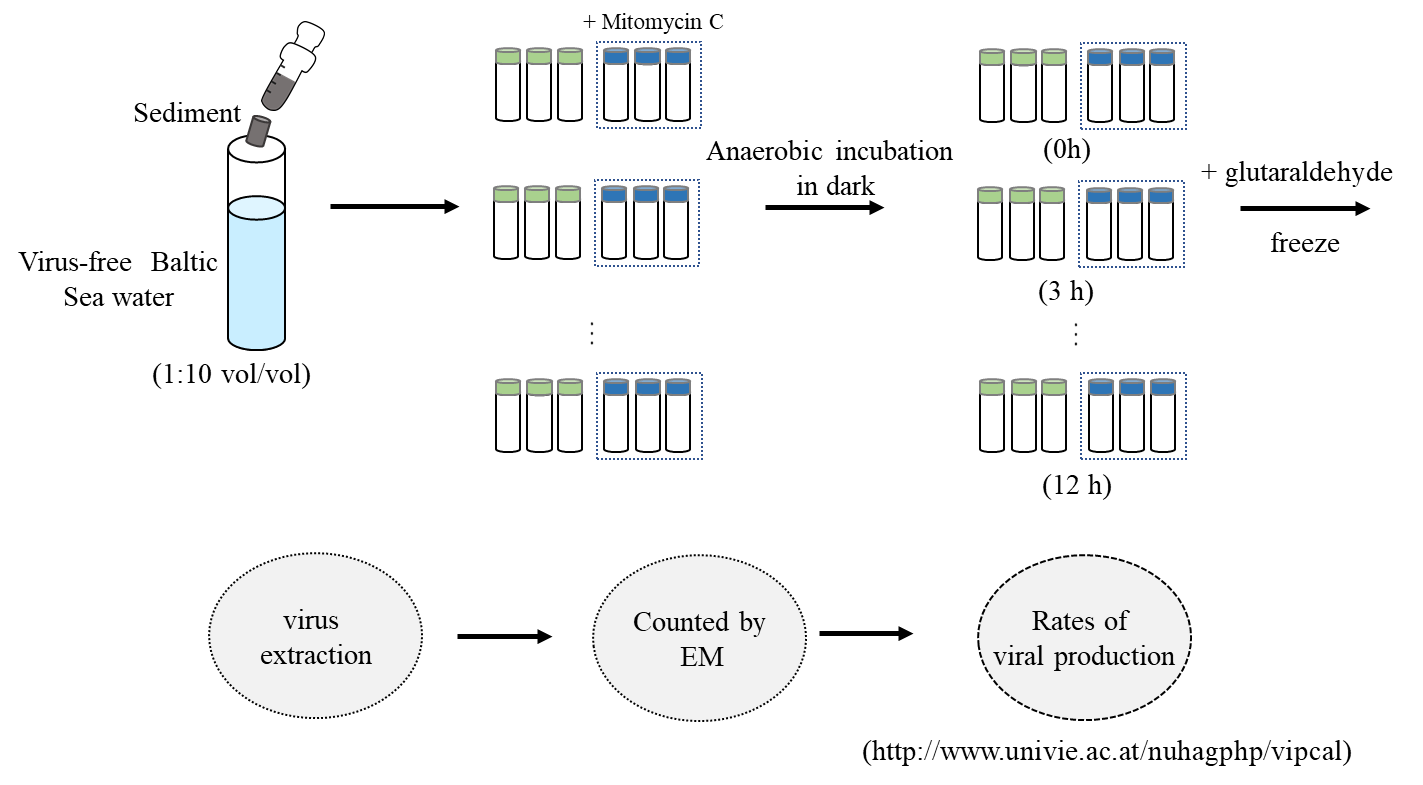


Fig. S3. Flow diagram showing the experimental procedure for estimating viral production in the sediment. EM: Epifluorescence Microscopy.


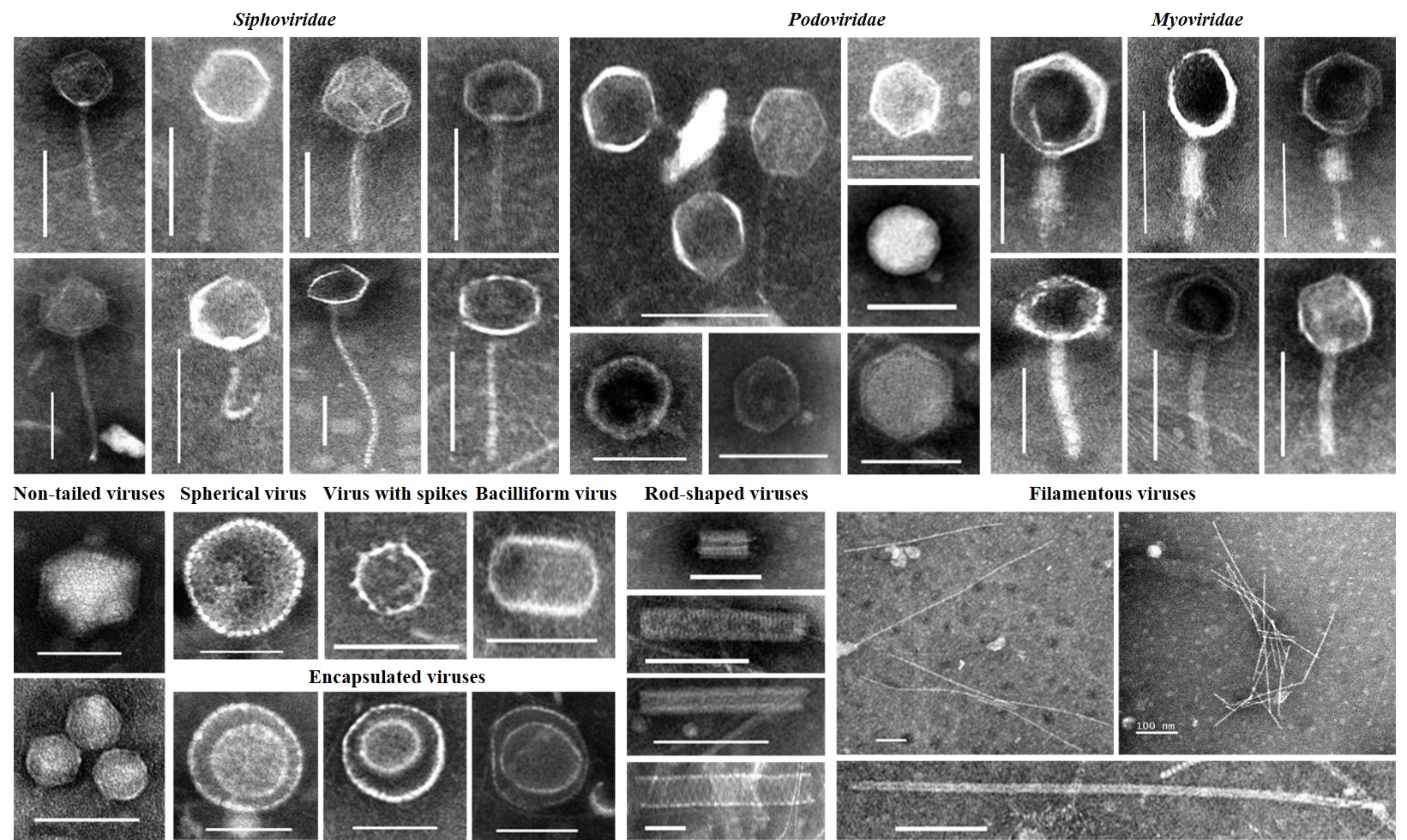


Fig. S4. TEM micrographs showing morphologies of virus-like particles observed in sediments of the Baltic Sea. Scale bar: 100 nm.


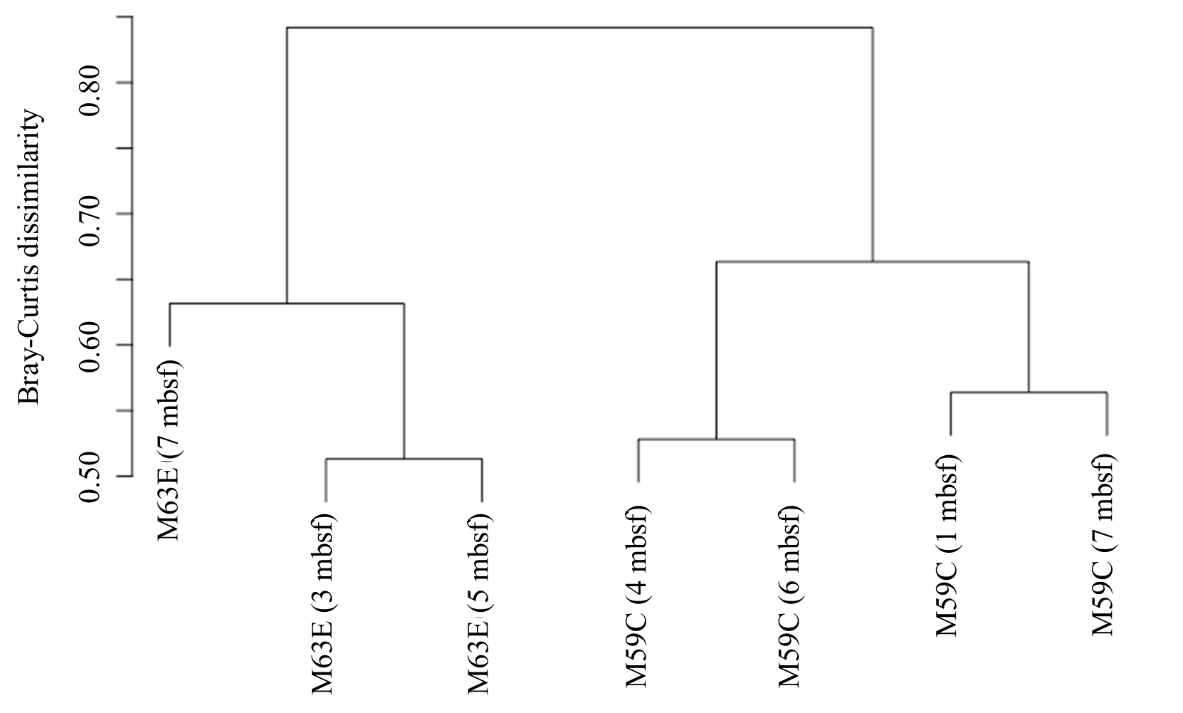


Fig. S5. Dendrogram illustrating hierarchical clustering of seven sediment samples from holes M59C and M63E. Samples are clustered using a Bray-Curtis dissimilarity matrix for all *g23* OTUs.


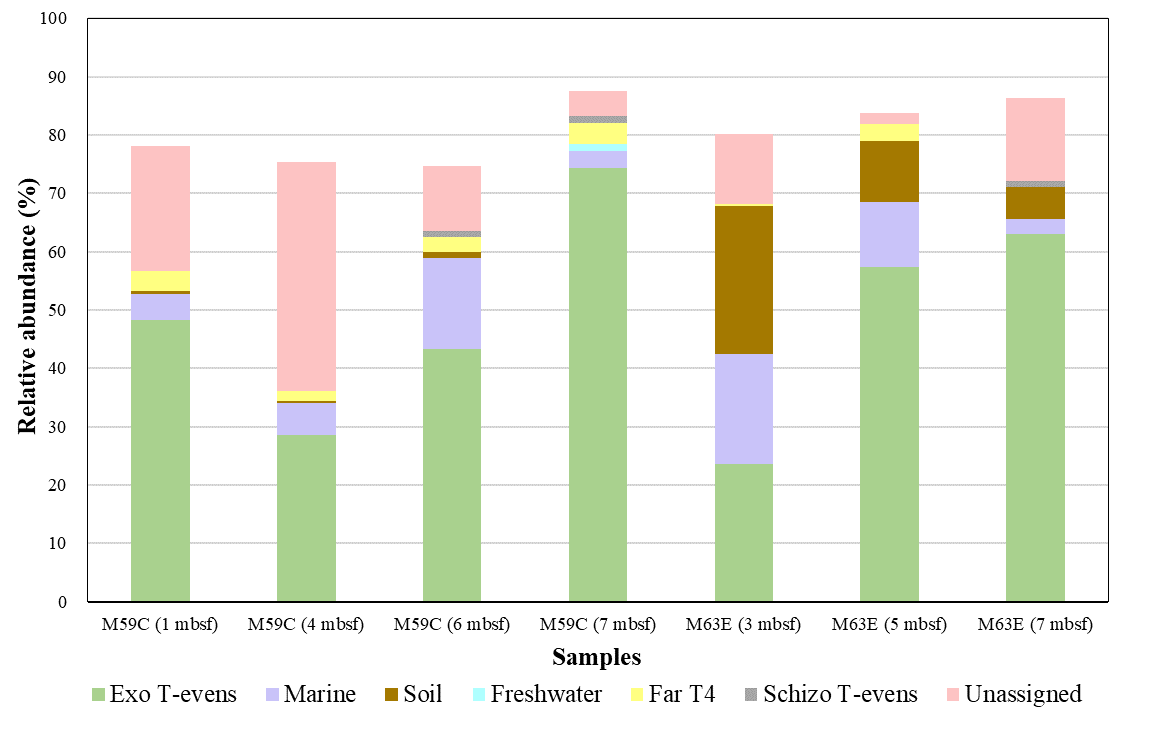


Fig. S6. The distribution of *g23* sequences of major OTUs in different sub-clusters in each sample. Different colors indicate *g23* sequences from different groups or origins. Yellow indicates the Far-T4 group, a neighbor to the clade that includes T4-like phages. Green indicates Exo-T evens, a group consisting of T4-like viruses infecting cyanobacteria. Gray indicates Schizo T-evens, a sub-group of T4-like phages. Sequences that originate from freshwater are in light cyan, and from soil in brown. Pink indicates sequences without assigned relatives.

Table S1. Pair-wise distances between samples using a Bray-Curtis dissimilarity analysis for all *g23* OTUs.

|  | 59C  1 mbsf | 59C  4 mbsf | 59C  6 mbsf | 59C  7 mbsf | 63E  3 mbsf | 63E  5 mbsf |
| --- | --- | --- | --- | --- | --- | --- |
| 59C_4 mbsf | 0.7209 |  |  |  |  |  |
| 59C_6 mbsf | 0.8264 | 0.5731 |  |  |  |  |
| 59C_7 mbsf | 0.5104 | 0.8365 | 0.8196 |  |  |  |
| 63E_3 mbsf | 0.9351 | 0.9488 | 0.9593 | 0.9562 |  |  |
| 63E_5 mbsf | 0.5981 | 0.9388 | 0.9386 | 0.5841 | 0.6597 |  |
| 63E_7 mbsf | 0.9592 | 0.9719 | 0.9781 | 0.9719 | 0.6975 | 0.8427 |

Table S2. BIOENV correlation coefficients between one or more environmental variables and the viral genetic composition based on *g23* sequences.

| **Variable** | ***r*** | ***p*** |
| --- | --- | --- |
| ***Ranked single factors*** |  |  |
| Rb | 0.6831 | 0.0030 |
| Li^+^ | 0.6481 | 0.0050 |
| Ca^2+^ | 0.6052 | 0.0150 |
| K^+^ | 0.5701 | 0.0130 |
| Sr^2+^ | 0.5429 | 0.0190 |
| Salinity | 0.5364 | 0.0220 |
| Na^+^ | 0.5364 | 0.0220 |
| Mg^2+^ | 0.5325 | 0.0240 |
| Br^-^ | 0.5286 | 0.0210 |
| TOC | 0.5000 | 0.0330 |
| Cl^-^ | 0.4948 | 0.0200 |
| VPR | 0.4390 | 0.0380 |
| Alkalinity | 0.4208 | 0.0490 |
| ***Best match of all factors*** |  |  |
| VA, PA, VPR, Sr^2+^, H_4_SiO_4_, PO_4_, Fe^2+^, Rb | 0.7416 | 0.0056 |

TOC: total organic carbon in the sediment; VPR: virus-prokaryote ratio; VA: viral abundance; PA: prokaryotic abundance. The data for environmental parameters is from IODP 347 Expedition Reports and is available online (<http://publications.iodp.org/proceedings/347/347toc.htm>).
